# Supplementary material for: A general theoretical framework to design base editors with reduced bystander effects
Source: Nat Commun. 2021 Nov 11;12:6529. doi: 10.1038/s41467-021-26789-5 (PMC8586357; doi:10.1038/s41467-021-26789-5)

Supplementary Information Appendix

To obtain the analytical solution of the editing probability for the kinetic model in Fig. 1, we introduced a first-passage probability density function $F_i(t)$, representing the probability to finish the editing process at time *t*, starting from the state *i*. Then the editing probability can be calculated as

$$P = \int_0^\infty F_0(t)dt \quad \text{[S1]}$$

The temporal evolution of $F_i(t)$ is controlled by the backward master equations:

$$\frac{dF_0(t)}{dt} = u_0F_2(t) + u_4F_1(t) - (u_0 + u_4)F_0(t) \quad \text{[S2]}$$

$$\frac{dF_2(t)}{dt} = u_1F_3(t) + u_2F_4(t) + w_0F_0(t) - (u_1 + u_2 + w_0)F_2(t) \quad \text{[S3]}$$

$$\frac{dF_3(t)}{dt} = u_3F_5(t) + w_1F_2(t) - (u_3 + w_1)F_3(t) \quad \text{[S4]}$$

$$\frac{dF_4(t)}{dt} = u_3F_6(t) + w_2F_2(t) - (u_3 + w_2)F_4(t) \quad \text{[S5]}$$

$$\frac{dF_5(t)}{dt} = u_2F_9(t) + w_0F_7(t) - (u_2 + w_0)F_5(t) \quad \text{[S6]}$$

$$\frac{dF_6(t)}{dt} = u_1F_{10}(t) + w_0F_8(t) - (u_1 + w_0)F_6(t) \quad \text{[S7]}$$

$$\frac{dF_7(t)}{dt} = u_4F_{13}(t) + mu_0F_5(t) - (u_4 + mu_0)F_7(t) \quad \text{[S8]}$$

$$\frac{dF_8(t)}{dt} = u_4F_{14}(t) + mu_0F_6(t) - (u_4 + mu_0)F_8(t) \quad \text{[S9]}$$

$$\frac{dF_9(t)}{dt} = u_3F_{11}(t) + w_2F_5(t) - (u_3 + w_2)F_9(t) \quad \text{[S10]}$$

$$\frac{dF_{10}(t)}{dt} = u_3F_{11}(t) + w_1F_6(t) - (u_3 + w_1)F_{10}(t) \quad \text{[S11]}$$

$$\frac{dF_{11}(t)}{dt} = w_0F_{12}(t) - w_0F_{11}(t) \quad \text{[S12]}$$

Eqns. [S1-S12] can be solved by utilizing Laplace transformations:

$$F_i(s) = \int_0^\infty F_i(t)e^{-st}dt \quad \text{[S13]}$$

Then eqns. [S1-S12] can be transformed to linear equations:

$$(s + u_0 + u_4)F_0(s) = u_0F_2(s) + u_4F_1(s) \quad \text{[S14]}$$

$$(s + u_1 + u_2 + w_0)F_2(s) = u_1F_3(s) + u_2F_4(s) + w_0F_0(s) \quad \text{[S15]}$$

$$(s + u_3 + w_1)F_3(s) = u_3F_5(s) + w_1F_2(s) \quad \text{[S16]}$$

$$(s + u_3 + w_2)F_4(s) = u_3F_6(s) + w_2F_2(s) \quad \text{[S17]}$$

$$(s + u_2 + w_0)F_5(s) = u_2F_9(s) + w_0F_7(s) \quad \text{[S18]}$$

$$(s + u_1 + w_0)F_6(s) = u_1F_{10}(s) + w_0F_8(s) \quad \text{[S19]}$$

$$(s + u_4 + mu_0)F_7(s) = u_4F_{13}(s) + mu_0F_5(s) \quad \text{[S20]}$$

$$(s + u_4 + mu_0)F_8(s) = u_4F_{14}(s) + mu_0F_6(s) \quad \text{[S21]}$$

$$(s + u_3 + w_2)F_9(s) = u_3F_{11}(s) + w_2F_5(s) \quad \text{[S22]}$$

$$(s + u_3 + w_1)F_{10}(s) = u_3F_{11}(s) + w_1F_6(s) \quad \text{[S23]}$$

$$(s + w_0)F_{11}(s) = w_0F_{12}(s) \quad \text{[S24]}$$

In addition, the editing probability can be written as:

$$P = F_0(s)|_{s=0} \quad [S25]$$

Solving the editing probability at different states requires different boundary conditions. As detailed in the main text, there are a total of four editing possibilities: CTC (Fig. 1, state 1, failed editing), CTT (state 13, only the target base is edited), TTC (state 14, only the is bystander edited) and TTT (state 12, both the target and the bystander base are edited).

For CTC, the boundary conditions are:

$$F_1(s) = 1;\ F_{12}(s) = 0;\ F_{13}(s) = 0;\ F_{14}(s) = 0 \quad [S26]$$

For CTT:

$$F_1(s) = 0;\ F_{12}(s) = 0;\ F_{13}(s) = 1;\ F_{14}(s) = 0 \quad [S27]$$

For TTC:

$$F_1(s) = 0;\ F_{12}(s) = 0;\ F_{13}(s) = 0;\ F_{14}(s) = 1 \quad [S28]$$

For TTT:

$$F_1(s) = 0;\ F_{12}(s) = 1;\ F_{13}(s) = 0;\ F_{14}(s) = 0 \quad [S29]$$

Solving P from eqns. [S14-S29] gives the analytical solutions of the editing probability (eqns. [1-5] in the main text).

We note that due to lack of experimental data, our model simplifies the influence from residue mutations. Strictly speaking, the energy perturbation due to mutations also mildly influences the on-rate besides the off-rate. So that eqns. [8-9] in the main text generally should be written as

$$u_2 = u_1 e^{\theta \cdot \Delta\Delta E_0/kT} \quad [S30]$$

$$w_2 = w_1 e^{(1-\theta) \cdot \Delta\Delta E_0/kT} \quad [S31]$$

while θ is a distribution factor ($0 < \theta < 1$). However, this approximation will not qualitatively change the main conclusions of this work. A more detailed theoretical model can be easily implemented when more experimental data are available. In addition, it is important to add that our theoretical method could also evaluate the reaction times for the editing, which can be viewed as mean first-passage times in our approach. The editing time can be calculated as:

$$T = -dF_0(s)/ds|_{s=0}/P \quad [S32]$$

With future advances in experimental methods this might also serve as another way of determining the parameters of the system.

Supplementary Table 1. Calculations for all A3A mutations in Figure 3. $\Delta\Delta E_m$ represents the binding free energy changes due to mutations, calculated by simulations. The unit of energy is $k_B T$. R_2 represents the probability ratio between CTT and TTT, calculated by the analytical model.

	WT	S99A	Y130F	N57Q	N57G
$\Delta\Delta E_m$	0	0.9±0.24	1.97±0.18	3.35±0.42	4.45±0.26
R_2	0.17	0.42	1.23	4.91	14.76

Supplementary Table 2. Genomic loci for Sanger sequencing. Forward and reverse primers for genomic DNA amplification are listed. The spacer sequences with PAM (underlined) are also listed with bystander C labeled red.

Genomic Loci	primers ID	Sequences (5'-->3')	Spacers + PAM
EMX1 #a3	EMX1#a3_F	CCTTTCCTGCTCTGGAATGTT	GAGTCCTGTGTGGGAGGAT GAGG
	EMX1#a3_R	CAGCTTTCTCTCTTCCCTGG	
	EMX1#a3_S	GTGACCTGAATTTTAGGGAA	
FANCE #1	FANCE_F	CCAGAGACAGCTCCAAAGTC	GGATTCCAGTGACTTATGTT CGG
	FANCE_R	CTACAGTCAACTCCATTGGGAAA	
	FANCE_S	CACCACTGCTGAAAGATCAC	
MRPL40#1	MRPL40_F	CACCTACACAGTTCTCACAAGT	GAAGATCCGAAAACTGGAA AAGG
	MRPL40_R	ACCAATGACTATCACAGGTGTG	
	MRPL40_S	ATGAGTTAGTCATGCACAGC	
MMS22L#1	MMS22L_F	CCCAAATTTTGGGGAATAAGAAAA TAAATC	gTTATCCAGGATTATGGTAT GAGG
	MMS22L_R	GTCATGGCTCAACTTAAATAAGCC	
	MMS22L_S	AAATTTTATAATCACTGCCCCC	
MRPL44 #1	MRPL44_F	TACTTTCGTTCCGTCTTCCATC	gGCTTCCAGAAGGAGTTAGA GCGG
	MRPL44_R	TTCTCCCAATTTTCAGTAGCCAA	
	MRPL44_S	GTCTCCATCGCAAACTTCCT	
FANCF#c1	FANCF_F	CAGGAGGACTCTCTGATGAAGA	gCTTCCTGAAGGTGATAGCG GTGG
	FANCF_R	CTGACAGAGGCTTTGAAACCTA	
	FANCF_S	TCACTAAAGTCAAAAGCCCG	
PPP1R12C #a1	PPP1R12C#a 1_F	TAACAGGTACCATGTGGGGT	gAGATCCAGGGACACGGTG CTAGG
	PPP1R12C#a 1_R	CGATATCTAGGTAGCCACAGGA	
	PPP1R12C#a 1_S	TCACTTCCTGTTTGCAGATA	
ATM #1	ATM_F	AGTCCGAAGAAGAGAAGCATTT	GTGATTCCTTGAGATTCTGG TGG
	ATM_R	TGTTTTTCCTGCCCCTATTTCT	
	ATM_S	GCAAAGCATTAGGTACTTGGTT	

F: forward primer; R: reverse primer; S: sanger sequencing primer; PAM: protospacer adjacent motif; bystander

Supplementary Figure 1. Calculated editing probability is affected by parameter m (0<m<1). m = 0 means that successful editing abolishes re-binding of Cas9 to ssDNA due to sgRNA mismatch. m = 1 means that successful editing has no effect on re-binding of Cas9. $\Delta\Delta E_m$ represents the binding free energy perturbations due to different mutations. The unit of energy is $k_B T$. P_t and P_b are the overall probabilities of editing the target and bystander cytidine, respectively. Source data are provided as a Source Data file.

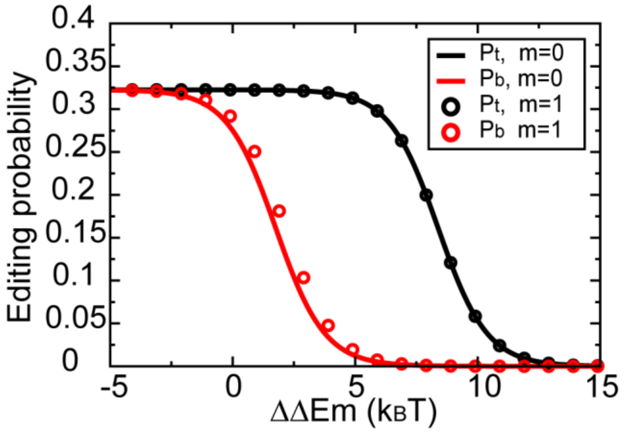

Supplementary Figure 2. A3G mutants editing the *EMX1* site 1. **a** Experimental measurements and **b** theoretical calculations. Bar plots represent mean ± s.e.m. (standard error of mean) of three independent biological replicates. P values were analyzed using two-tailed Student's t-test. Source data are provided as a Source Data file.

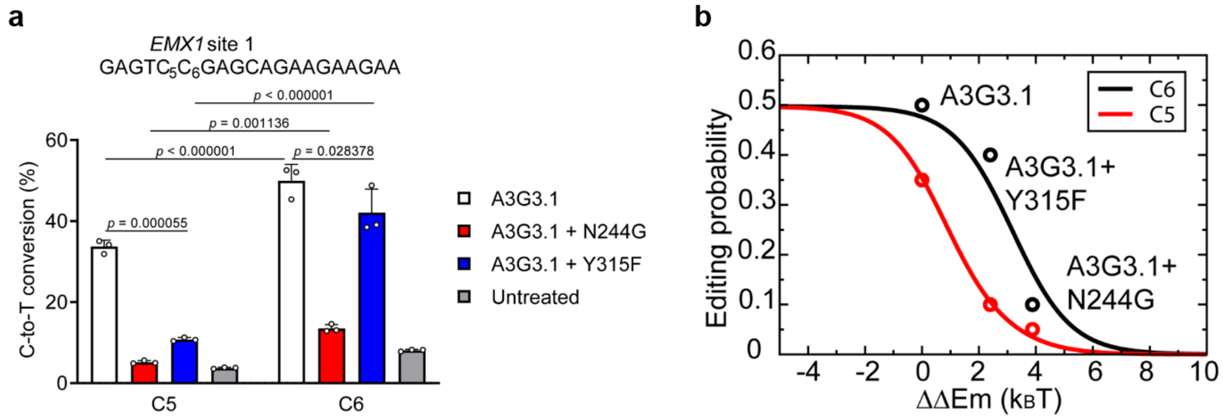

Supplementary Figure 3. Theoretical model reproducing the editing patterns of six genomic loci observed in experiments (ref [11]). The cell type is human HEK293T except the last one is erythroid precursor cell. Source data are provided as a Source Data file.

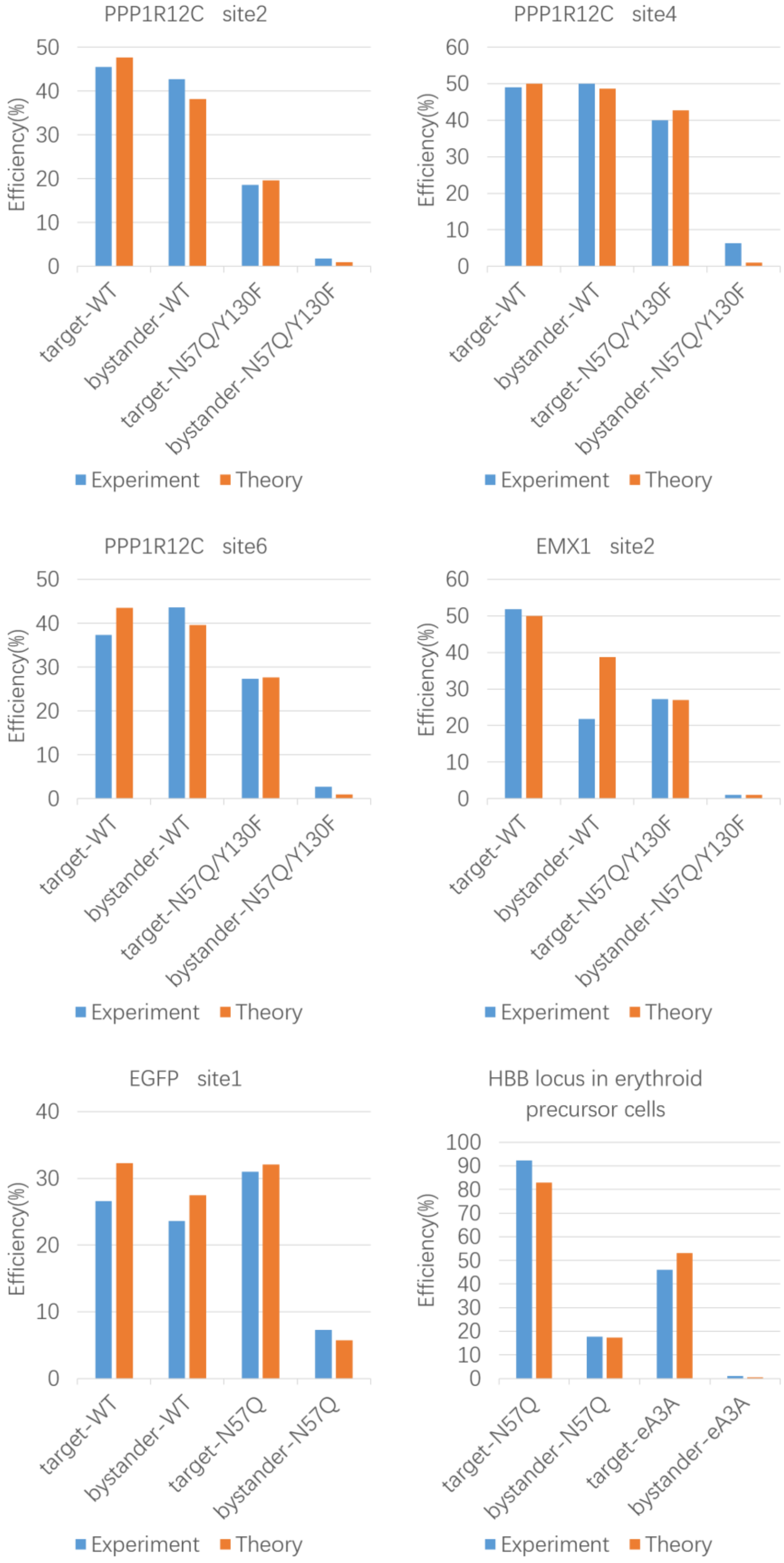

Supplementary Figure 4. Experimental measurements at EMX1 #a3 site for A3G3.1, A3G3.14 (T218S) and A3G3.15 (T218N) using three cell lines, **a** K562, **b** Jurkat, and **c** HeLa. Bar plots represent mean of two independent biological replicates, except for the bars representing the editing efficiency of A3G3.1, A3G3.15 and Untreated in HeLa cells, which shown the mean ± s.d. from three biological replicates. Two-tailed *p* values calculated from unpaired t-tests (A3G3.15 versus A3G3.1) were 0.0005, 0.0001 and 0.3786 for K562, Jurkat and Hela cells, respectively. Source data are provided as a Source Data file.

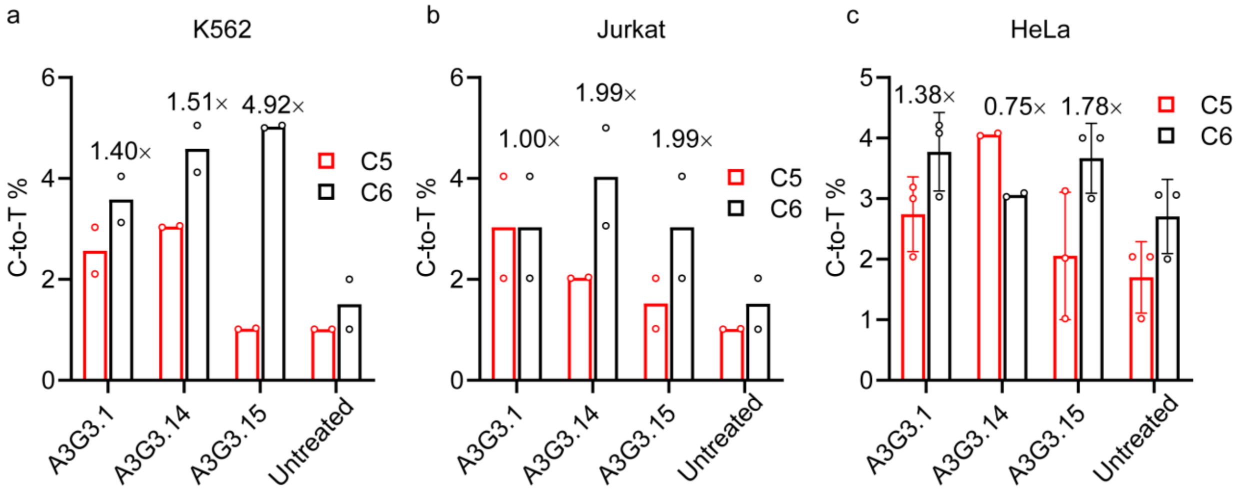

Supplement: Supplementary file 1 — Supplementary Information [file 41467_2021_26789_MOESM1_ESM.pdf]
