# Supplementary material for: A general theoretical framework to design base editors with reduced bystander effects
Source: Nat Commun. 2021 Nov 11;12:6529. doi: 10.1038/s41467-021-26789-5 (PMC8586357; doi:10.1038/s41467-021-26789-5)
Supplement: Supplementary file 2 — Reporting Summary [file 41467_2021_26789_MOESM2_ESM.pdf]

nature portfolio

Corresponding author(s): Anatoly B. Kolomeisky

Last updated by author(s): Oct 20, 2021

Reporting Summary

Nature Portfolio wishes to improve the reproducibility of the work that we publish. This form provides structure for consistency and transparency in reporting. For further information on Nature Portfolio policies, see our Editorial Policies and the Editorial Policy Checklist.

Statistics

For all statistical analyses, confirm that the following items are present in the figure legend, table legend, main text, or Methods section.

n/a	Confirmed	
☐	☒	The exact sample size (n) for each experimental group/condition, given as a discrete number and unit of measurement
☐	☒	A statement on whether measurements were taken from distinct samples or whether the same sample was measured repeatedly
☐	☒	The statistical test(s) used AND whether they are one- or two-sided<br>*Only common tests should be described solely by name; describe more complex techniques in the Methods section.*
☒	☐	A description of all covariates tested
☒	☐	A description of any assumptions or corrections, such as tests of normality and adjustment for multiple comparisons
☐	☒	A full description of the statistical parameters including central tendency (e.g. means) or other basic estimates (e.g. regression coefficient) AND variation (e.g. standard deviation) or associated estimates of uncertainty (e.g. confidence intervals)
☐	☒	For null hypothesis testing, the test statistic (e.g. F, t, r) with confidence intervals, effect sizes, degrees of freedom and P value noted<br>*Give P values as exact values whenever suitable.*
☒	☐	For Bayesian analysis, information on the choice of priors and Markov chain Monte Carlo settings
☒	☐	For hierarchical and complex designs, identification of the appropriate level for tests and full reporting of outcomes
☒	☐	Estimates of effect sizes (e.g. Cohen's d, Pearson's r), indicating how they were calculated

Our web collection on statistics for biologists contains articles on many of the points above.

Software and code

Policy information about availability of computer code

Data collection	Simulation data was performed by an open source software Gromacs2019
Data analysis	Free energy calculation was performed by an open source software Gromacs2019; Amplicon sequencing data was processed by CRISPResso2 (https://github.com/pinellolab/CRISPResso2) ; Sanger sequencing data was analyzed by EditR (http://baseeditr.com/)

For manuscripts utilizing custom algorithms or software that are central to the research but not yet described in published literature, software must be made available to editors and reviewers. We strongly encourage code deposition in a community repository (e.g. GitHub). See the Nature Portfolio guidelines for submitting code & software for further information.

Data

Policy information about availability of data

All manuscripts must include a data availability statement. This statement should provide the following information, where applicable:

- Accession codes, unique identifiers, or web links for publicly available datasets
- A description of any restrictions on data availability
- For clinical datasets or third party data, please ensure that the statement adheres to our policy

All data generated or analysed during this study are included in this published article (and its supplementary information files). Targeted Amplicon sequencing data have been deposited at the Sequence Read Archive (SRA): https://www.ncbi.nlm.nih.gov/bioproject/PRJNA700693.

Field-specific reporting

Please select the one below that is the best fit for your research. If you are not sure, read the appropriate sections before making your selection.

- [x] Life sciences
- [ ] Behavioural & social sciences
- [ ] Ecological, evolutionary & environmental sciences

For a reference copy of the document with all sections, see nature.com/documents/nr-reporting-summary-flat.pdf

Life sciences study design

All studies must disclose on these points even when the disclosure is negative.

Sample size	Sample sizes were determined based on literature precedence for genome editing experiments.
Data exclusions	No data was excluded from the analysis.
Replication	All experimental findings were independently replicated for two to four times. Most of the experiments were performed independently three or four times. For different cell line editing assays, we did two independent replicates. All replication attempts were successful.
Randomization	All independent replicates were treated identically and all data from all experiments were included, thus randomization was not relevant.
Blinding	All cells used in this study were grown under identical conditions ot relevant; blinding was not performed.

Reporting for specific materials, systems and methods

We require information from authors about some types of materials, experimental systems and methods used in many studies. Here, indicate whether each material, system or method listed is relevant to your study. If you are not sure if a list item applies to your research, read the appropriate section before selecting a response.

Materials & experimental systems

n/a	Involved in the study	
x		Antibodies
	x	Eukaryotic cell lines
x		Palaeontology and archaeology
x		Animals and other organisms
x		Human research participants
x		Clinical data
x		Dual use research of concern

Methods

n/a	Involved in the study	
x		ChIP-seq
x		Flow cytometry
x		MRI-based neuroimaging

Eukaryotic cell lines

Policy information about cell lines

Cell line source(s)	HEK293T (CRL-3216), HeLa (CCL-2), K562 (CRL-3343), and Jurkat (TIB-152) cell lines were obtained from ATCC
Authentication	COAs were provided with cell lines from ATCC. No other authentication was performed.
Mycoplasma contamination	Cell lines were routinely tested for mycoplasma and the results were all negative.
Commonly misidentified lines (See ICLAC register)	No commonly misidentified cell lines were used in this study.
